# Exploring customers’ perceptions of food adulteration at bazaars and supermarkets in Dhaka, Bangladesh; a qualitative exploration

**DOI:** 10.1186/s12889-022-14933-9

**Published:** 2023-01-31

**Authors:** Dalia Yeasmin, Musa Baker, Abu-Hena Mostofa Kamal, Md Saiful Islam, Mahbubur Rahman, Peter J. Winch, Leanne Unicomb

**Affiliations:** 1 Environmental Interventions Unit, Infectious Diseases Division, International Centre for Diarrhoeal Disease Research (icddr,b), 68 Shaheed Tajuddin Ahmed Sarani, 1212 Dhaka, Bangladesh; 2 Khulna University of Engineering & Technology (KUET), Khulna, Bangladesh; 3 Kirby Institute, Global Health Program, University of New South Wales, Sydney, Australia; 4 Johns Hopkins Bloomberg School of Public Health, Baltimore, MD USA

**Keywords:** Adulterants, Customer perceptions, Information, Food safety, Food quality, Wet market, Supermarket

## Abstract

**Background:**

Food adulteration is an increasingly recognized global public health problem. In low- and middle-income countries like Bangladesh, adulteration is difficult to detect and respond to. We explored customers’ perceptions on food adulteration, perception of risk and connections between information, participant characteristics and patterns of adulterated food concerns that impact risk perception in urban Bangladesh.

**Methods:**

A formative study was conducted in Dhaka, between June and August 2015 at a supermarket and a wet market. We explored community awareness and response to chemical contaminants (adulterants) among participants from a range of socio-economic backgrounds*.* The team conducted 38 in-depth interviews with 12 customers and 4 staff from a supermarket, and 12 customers and 10 vendors from a wet market. Participants were selected purposively. Audio recorded data were coded based on thematic content and analyzed manually.

**Results:**

We asked participants how common foods were likely adulterated, and most gave figures of 70% or more. They reported that foods were adulterated with chemicals or artificial colors, especially fish, milk, and vegetables. The supermarket more commonly sold packaged foods with nutritional and expiry information on the label; and offered convenience in terms of building size, layout, and cleanliness. All customers from the wet market thought that foods were cheaper and fresher than from supermarkets. Supermarket customers expressed greater concern about adulterated foods than wet market customers. Most participants from both markets reported that food adulteration is invisible, adulterated foods cannot be avoided, and have long-term negative health impacts including cancer, diabetes, paralysis, heart attack, and others. Nearly half of customers from both markets were concerned about the poor nutritional value of adulterated food. Participants from both settings expressed the need for access to credible information about adulteration to help choose safe foods. The majority expressed the need for government action against those who are responsible for adulteration.

**Conclusions:**

Food adulteration was considered a major health threat. The government could act on food adulteration prevention if provided credible population-based data on disease burden, a model food sampling and testing protocol, a model for inspections, organizational strengthening and training, example social and behavioral change communications with estimated costs.

## Introduction

Worldwide, including in low- and middle-income countries (LMICs) like Bangladesh, documented food adulteration is a common and increasingly recognized public health problem with alarming consequences [1–6]. The consumption of adulterated foods has been estimated to cause health problems in approximately 57% of people globally [3, 7, 8]. In South Asia almost 150 million people suffer from diseases related to food adulteration where Bangladesh tops the list [9].

Resultant diseases depend on the food adulterant but range from cancer, diabetes, paralysis, kidney disease, blood disorders, bone marrow abnormality, heart disease, and skin problems including allergic manifestation [1, 3, 8–11]. Consumption of adulterated milk, fish, spices, onion, ginger, coriander, chili, turmeric, cumin by pregnant women can lead to abortion or fetal brain damage [12, 13]. Consuming adulterated foods can deprive the body of nutrients essential for growth and development can an impact a child’s mental health and physical growth [14, 15]. Food adulterants -di-(2-Ethylhexyl) phthalate (DEHP), diisononyl phthalate (DiNP), and melamine are harmful to the hippocampus, kidneys, reproductive organs, and immune system of children, and they can increase the risk of cancer [16]. A study conducted by the Institute of Nutrition and Food Science at Dhaka University and a further survey estimated that intake of adulterated foods and inadequate diets contribute to malnutrition [11, 17].

In Bangladesh, food adulteration is perceived as the greatest threat to food safety [18]. Recently, public concern about food adulteration has increased noticeably [19]. Adulteration occurs when substances are added to color, coat, preserve foods to enhance their perceived quality or quantity to increase profitability [20]. In addition, adulterants may be innocuous “filler” substances like grain flour added to spices to increase quantity, or highly toxic adulterants like industrial pigments, textile dyes added to spices to improve color and to increase acceptability [6, 21].

Multiple studies from Bangladesh have identified a range of foods- meat, edible oils, spices, bakery items sweetmeats that were adulterated with hazardous substances including toxic pesticides- Dichlorodiphenyltrichloroethane (DDT), toxic chemicals such as formalin, calcium carbide, formalin, sodium cyclamate, metanil yellow, urea, textile dyes or microbiological contamination among up to 50% of food items [10, 15, 22, 23]. The levels of toxic substances in these samples were up to 20 times the maximum set by the European Union [15]. The chemical adulterants added to food are perceived as riskier than microbiological contamination due to limited understanding of the risks, and few means to avoid exposure to substances produced in an industrial process when the perceived benefits are low [24].

A cross-sectional survey enrolling 96 adult male and female participants in eight Dhaka city corporation wet markets found that they perceived between 37 and 80% of food items as adulterated [23]. Essential food items like vegetables, fruits, fish, and chicken from three-fourths of markets contained formalin residues [3, 11]. A study reported that the overall proportion of adulterated food samples decreased (from 75 to 64%) from 2001 to 2005. Though adulteration has been reported and investigated extensively in Bangladesh [11, 25], there is limited information on the extent of the effects on human health or ways to address the public health burden [26].

The availability of reliable information about adulterants to enable customers to choose safer foods is limited [27]. It is important to understand public responses to risk information related to food adulteration [28]. Misconceptions and a lack of knowledge are associated with higher levels of concern related to foods and their adulteration in recent studies [29]. The common interpretation is that people cannot differentiate between toxicological hazards and risks, however, a prior study suggested that people understood the seriousness of adulteration and human health risk [24]. While people generally lack knowledge of risk assessment and regulation of food additives [30], they may still believe that there are health impacts of chemicals applied to food. This likely contributes to unfavorable attitudes towards chemicals in general, as well as unfavorable attitudes about food additives and food packaging [31]. Therefore, developing a more nuanced understanding of customers’ perceptions about food adulteration can inform the government of priorities among the general population to develop programs to reduce health burden [32].

The perceived credibility of information and attitudes about risk depend on scientific evidence and information sources [33]. Factors that potentially influence how individuals choose safe foods include the availability of trustworthy information, perception related to threat and ability to pay, age, gender, ethnicity, personality, socioeconomic status, and education level, and how an individual interprets information, motivation, and convenience [8, 34, 35]. Obtaining information on perceived reliable information sources can serve to inform methods of dissemination.

The Government of Bangladesh Food Safety Act, 2013 defines what is considered contaminated, adulterated, misbranded food, or food ingredients which do not comply with standards prescribed by the law. It states that information on food adulteration must be disclosed to the general public and the government should take necessary steps to withdraw the food or food ingredient from markets. The role of courts in food safety management is to punish offenders who violate laws contained in the Act. However, enforcement of the Government of Bangladesh Food Safety Act, 2013 has been limited.

Globally, food adulteration predominantly occurs in urban areas [36, 37]. In this study, we aimed to explore connections between information (where and what individuals purchase and how much they pay), participant characteristics, and patterns of adulterated food concerns among the urban population. Through this qualitative study, we sought to understand how individuals obtain information about adulterated foods, how they interpret that information, determine food adulteration-related perceptions, risk appraisal of these substances, and how they behave as customers at supermarkets selling certified formalin-free products and at wet markets. Gaining a deeper understanding of uncertain risks from consuming adulterated food, and customer purchase decision making can inform priorities for policy. Additionally, we explored potential health-supportive strategies to control food adulteration practices.

## Methods

### Study sites and participants

**Fig. 1:**
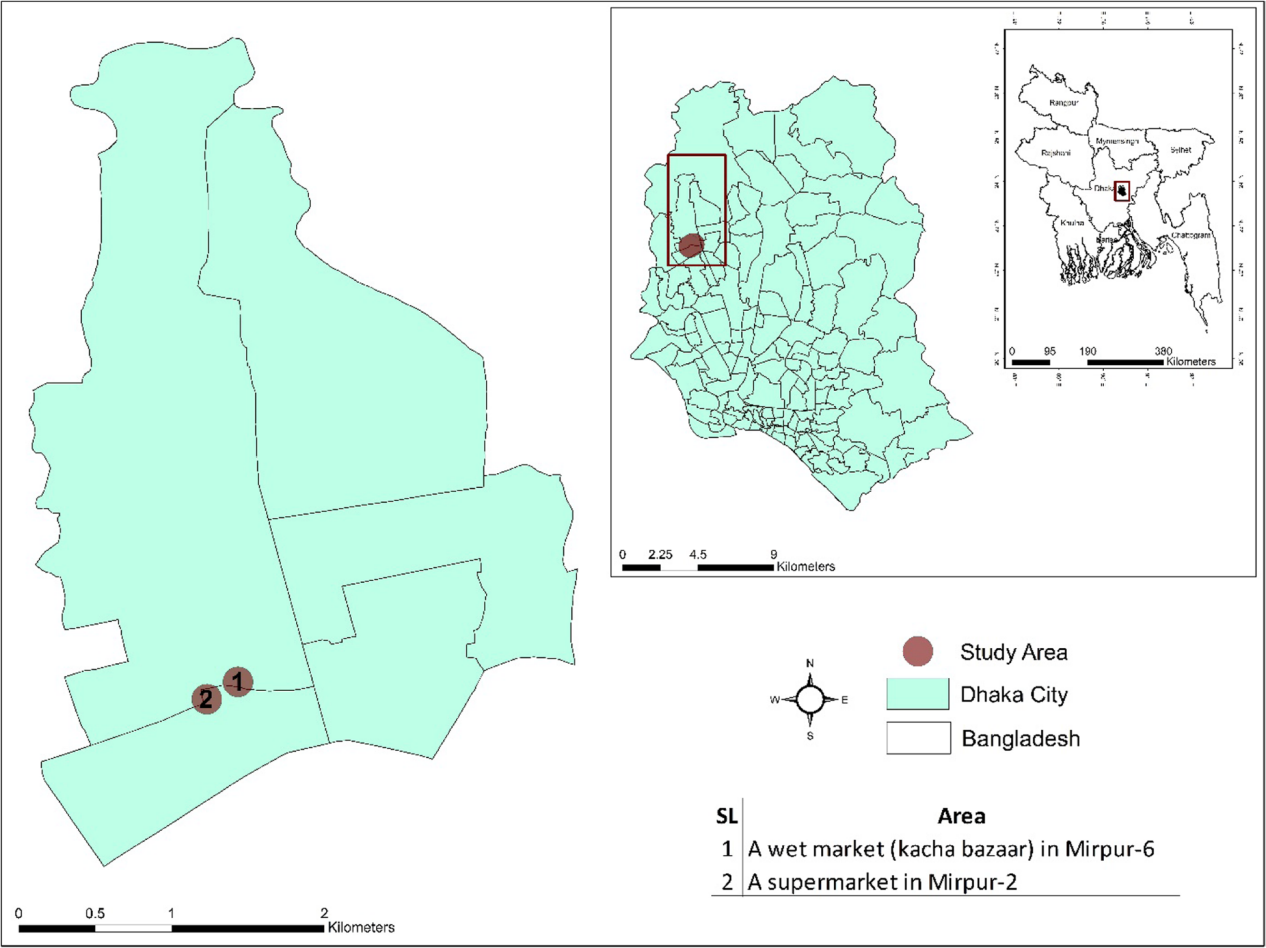
Study area map

We conducted a formative study (qualitative study) between June and August 2015 in the Mirpur area, in the capital city, Dhaka, Bangladesh (Table 1 and Fig. 1). The study site included two types of shopping venues: a wet market (*kacha bazaar*) in Mirpur-6 and a supermarket in Mirpur-2; both sold similar items including fish, meat, vegetables, fruits, rice, grocery, and cosmetics items. The wet market was larger in terms of range of goods sold by vendors and wholesalers, with around 3000 customer visits per day (Table 2). It comprised multiple independent stalls. The supermarket sold goods together in a single location, and sold certified formalin-free foods. The two sites are in two diverse socio-economic neighborhoods, chosen to explore differences in awareness and response to adulterants by socio-economic status*.* The wet market was located in a lower-income neighborhood while the supermarket was located in a higher-income neighborhood.

**Table 1:** Demographic characteristics of customers, vendors and shop staff at wet market and supermarket in Dhaka, Bangladesh, June & August 2015

Characteristic of participants	customers n (%) (***N*** = 24)	vendors & shop staff n (%) (***N*** = 14)
**Category of interviews**		
Customers		
Wet market	12 (50)	**–**
Supermarket	12 (50)	**–**
Rice vendors/staff		
Wet market	**–**	4 (29)
Supermarket	**–**	1 (7)
Fish vendors/staff	**–**	
Wet market	**–**	3 (22)
Supermarket	**–**	1 (7)
Wholesalers (supermarket)	**–**	2 (14)
Assistant outlet Operation Manager	**–**	1 (7)
Chief Operation Officer	**–**	1 (7)
Market committee members	**–**	1 (7)
**Household primary caregivers**		
Male	16 (67)	10 (71)
Female	8 (33)	4 (29)
**Age of participants (years); mean [range]**	29 [19–60]	24 [20–52]
**Educational qualification**		
No formal Education	2 (8)	–
Primary	10 (42)	5 (36)
Higher	12 (50)	9 (64)
**Mean Household size**	4	5
**Profession**		
Homemaker	4 (17)	–
Business	4 (17)	4 (29)
Service	14 (58)	10 (71)
Rickshaw puller	2 (8)	–
**Household monthly income in BDT**^**a**^ **(USD)**		
< 10,000 (117)	2 (8)	–
10,000–20,000 (117–335)	10 (42)	2 (14)
21,000–30,000 (246–352)	6 (25)	4 (29)
31,000–40,000 (363–467)	4 (17)	4 (29)
> 41, 000 (480)	2 (8)	4 (29)
**Religion**		
Muslim	20 (83)	12 (86)
Hindu	3 (13)	2 (14)
Christian	1 (4)	–
**Number of family members**		
1–2	2 (8)	2 (14)
3–4	12 (50)	8 (57)
5–7	10 (42)	4 (29)
**Types of interviews**		
In-depth interview	24 (100)	13 (93)
Group discussion	–	1 (7)

^a^*BDT* Bangladeshi taka

**Table 2:** Physical environment and observed characteristics for the wet market and the supermarket in Dhaka, Bangladesh between June and August 2015

Market name	Distance from main road	Items for sale	Number of customer visit per day	Customer gender	Typical customer characteristics
Wet market, Mirpur-6	Within 1 m	-All grocery items (both in lose form and some pre-packaged -Chicken (dressed and live) -Meat -fish (live) -Rice (both packaged and lose form, mostly sold in bulk amount) -Fruits, vegetables (most foods were non-prepackaged and without label) -cosmetics (fewer items than the supermarket)	2500–3000	male (7 of 12 customers)	-Service holder -Day laborer -homemaker
Supermarket, Mirpur-2	Within 1 m	-All grocery items-mostly in packaged form and labelled -fish (fresh fish, prepackaged form ready for cooking) -Chicken, meat -Rice (pre-packaged) - Fruits, vegetables (people choose) -cosmetics (common national brands)	1200–1500	Male (6 of 12 customers)	-Service holder -Businessman -Homemaker

For the formative study, we purposively selected customers and vendors from both markets for in-depth interviews and group discussions based on availability, and willingness to participate. The team visited once in the morning, afternoon, and evening therefore three different periods to select individuals for interviews to diversify participants. The team selected both male and female participants for each of the shopping periods to determine differences in perceptions along with the understanding of adulterated foods, pattern of food adulteration perceptions, and to ensure broad data variation. The interviewers determined eligibility before data collection and obtained informed consent.

### Data collection

The formative study enrolled 38 participants from the two markets for qualitative exploration which included in-depth interviews with 12 customers and 4 staff from a supermarket, 12 customers and 9 vendors from a wet market, and finally a group discussion with market committee members from the wet market (Table 1). Customers were eligible if they were the primary shopper for their family, not household staff shopping on behalf of someone else. The team conducted in-depth interviews with vendors from a wet market and staff from a supermarket that sold rice or fish to understand behaviors, strategies to improve food adulteration and inform policies to improve the quality of foods available at markets. Rice sellers were eligible if they sold *najirshail* or *minicat* (commonly purchased by higher-income individuals) or BR 28 rice varieties (commonly purchased by lower-income individuals). Initially, the team conducted and coded 5 in-depth interviews to assess data trends and variability among participants before continuing further interviews where data saturation occurred after 12 in-depth interviews.

We selected participants purposively to identify their perceptions and behaviors of adulterated foods (where and what individuals purchase and how much they pay) and how these perceptions impact food purchasing behavior. For in-depth interviews, the team purposively selected nine vendors from the wet market who were involved in selling goods for 6–10 years; five were rice vendors- and four were fish vendors- and two were rice wholesalers. From the supermarket, the team selected one supermarket Assistant Operations Manager and the supermarket Chief Officer (Table 1). We explored perceptions of food adulteration, food quality, food safety, the impact of food adulteration on health, and preventative strategies to control food adulteration practices. To capture the opinions of the wet market’s committee members (*N* = 11) we conducted a group discussion to explore perceptions and access to credible information on adulterated foods.

### Data analysis

The data were collected by the first author, who is an anthropologist, along with two sociologists, who had extensive experience collecting both qualitative and quantitative data. Before data collection commenced, the team developed data collection guidelines and coding guides based on research objectives.

Audio recorded data from both in-depth interviews and group discussions were transcribed verbatim in Bengali then translated into English. Based on themes developed a priori and emergent themes, we created codes based on the study objectives. Transcripts were manually coded and categorized according to these major codes. We then coded the data based on thematic content. Additional field notes from informal discussions and observations included non-verbal cues and attitudes of the participants during data collection; these data were transcribed to generate additional relevant codes and themes. We analyzed each in-depth interview separately and in the findings, we have drawn inferences collectively as well as comparisons for socio-economic status, market type, pattern of goods sold and gender. Two coders of different backgrounds analyzed each interview to ensure accurate data interpretation.

## Results

### Participant characteristics

Of the 38 participants, 24 were customers who participated in in-depth interviews and 14 were vendors or shop staff; among 24 customers, 16 were male and among 14 vendors or shop staff, 10 were male (Table 1). Among customers, the average household size was 4 persons and the average participant age was 29. For vendors and shop staff, the household size was 5 persons, and the average mean age was 24. The majority (*n* = 29) of the participants had a family that included children and elders. Half of the customers completed at least secondary education whereas 64% of vendors/shop staff completed secondary education or above. Fifteen percent of customers from both markets (*n* = 6) had household income of approximately 21,000 Bangladesh Taka (BDT or USD 250)/ month and 42% of vendors/ staff from both markets earned less than 30,000 BDT (USD 353)/ month. One-quarter (24%) of the participants shopped for household foods daily, whereas the majority shopped once a week.

We summarize participant interview and discussion opinions and themes on understanding of adulterant risks; perceptions of food adulteration; sales and purchase of adulterated foods; factors influencing food purchase; and sources of reliable food adulteration information.

### Consumer understanding of chemical substances risks in foods

Most participants equated the term ‘chemical substance’ with a ‘harmful substance’. Notably, only a few participants conceptualized the harmfulness of a chemical in line with scientific understanding; the amount needed to cause adverse effects. Instead, they often defined harmfulness by the number of people affected by the chemical or the severity of the effects a chemical can cause diseases. Most participants expressed negative attitudes toward chemical substances in food in terms of potential harm.

### Perceptions of food adulteration

Most participants [34], when asked how commonly they thought food was adulterated with harmful chemicals or artificial colors gave figures of 70% and above. Based on information from television, family members, newspapers, other media, Facebook, friends, neighbors, colleagues, fellow customers, participants believed that food items such as spices, fish, vegetables, milk and milk products, rice, edible oils, and sweetmeats were most commonly adulterated and to varying degrees. They reported that non-nutritious and toxic substances such as formalin, calcium carbide, melamine, textile dyes, burnt engine oil, palm oil, alum powder, wax (to polish), urea, metanil yellow powder are added intentionally. Vendors (*n* = 4) and customers (*n* = 14) perceived that this problem persists at all levels of the food chain from preparation to consumption.

A customer from the supermarket said,

“Whatever we eat I think adulteration is there. Once I heard about the issue that in Dhaka 90% of the foods people consume are adulterated foods. These are adulterated in multiple ways, as a result, various diseases are increasing day by day, no one is safe, I am also included there. Now the thing is to me how can we overcome this dangerous situation?”

Most supermarket customers were concerned about adulterated foods (*n* = 10) and reported that there is no place to purchase unadulterated foods. Moreover, they do not know what the contamination status is, as the available information is inadequate (Table 3). They added that if they were to know which foods and vendors could ensure that the foods are free of adulteration they would pay more. In contrast, we found that half of the customers from the wet market (*n* = 6) were aware of food adulteration. Consequently, the cheaper price of foods were perceived greater longevity and attractive appearance were drivers of food purchase among customers from both markets (Table 3). Participants provided an extensive list of foods and the adulterants that they perceived were common for those foods. A range of industrial chemicals and components were described for foods that are frequently consumed (Table 4).

**Table 3:** Reasons customers and vendors purchase adulterated foods

	(***N*** = 38), n (%)
**Not aware about adulterated foods (as the available information to customers is inadequate)**	9 (24)^a^
**Cheaper price**	9 (24)^a^
**It has greater longevity**	7 (18)
**It looks nice**	5 (13)
**Pattern of food consumption**	3 (8)
**Others (better taste, habits)**	3 (8)
**Unavailability of pure (unadulterated) foods.**	2 (5)^a^

^a^ **=** Included multiple responses

**Table 4:** List of foods with adulterants and the purpose perceived by participants

Food item	Adulterants	Purpose	Number of participants & type of interview
**Fish, meat**	Formalin, chemicals	To look fresh, improve appearance, and preserved for long run, sell rotten	36^a^/IDIs & GD
**Chicken**	Spray in chicken with formalin, dead chicken	To keep it fresh for a loger duration, increase the quantity & maximize the profit	36^a^/ IDIs & GD
**Fruits –mango, apple, orange, grapes**	Calcium carbide	To ripen fruits and looks fresh	36^a^/ IDIs & GD
**vegetables-carrots, beans, tomatoes, green bananas**	Toxic substances, Calcium carbide	To keep fresh, maximize the profit, and for artificial ripening	36^a^/ IDIs & GD
**Milk-powdered milk**	Formalin, melamine, textile dyes	To increase the quantity & maximize the profit	36^a^/ IDIs & GD from IDIs & GD
**Sweetmeats**	Burnt engine oil, textile dyes	To enhance the color and looks attractive	36^a^/ IDIs & GD
**Rice**	Toxic substances	To increase the color, quality	24^a^/ IDIs & GD
	Alum powder	To lengthen the storage period	12^a^/ IDIs
	Used wax to polish	To make grains appear whiter with a silky surface	12^a^/ IDIs &
	Urea	To make it whiter	24^a^/ IDIs
**Edible oil/cooking oil** **Mustard oil**	Mixed with palm oil, toxic substances Chemicals	To improve moisture, Traders gain profit	12^a^/ IDIs
**Spices**	Artificial colors, toxic substances	To improve the appearance and color	19^a^/ customers IDIs & GD
**Puffed rice** ***(Muri)***	Urea	To lengthen the storage period	14 IDIs & GD
**Chili powder**	Brick dust, Sudan red	To improve the appearance, color, and quality	14/ IDIs
**Sauces, juices, and lentils**	Various coloring agents, chemicals	To increase the color	12/IDIs
**Turmeric powder**	Metanil yellow powder	To improve the color of turmeric	12/ IDIs customers
**Dry fish**	Dichlorodiphenyl tricholoroethane (DDT)	To keep it fresh	4/ IDIs
**Shrimp**	Sold rotten	To increase the profit	17/IDIs

^**a**^ **=** Included multiple responses

Participants reported that adulteration occurred to enhance food quality, improve appearance, texture, or food quantity. In addition, almost all of the participants said that adulteration occurs to maximize profit. Moreover, those aware of adulteration and risk expressed certainty about poor food safety, and uncertainty about acceptable risk.

### Sales and purchase of adulterated foods

Vendors/staff from both markets argued that wholesalers use various hazardous chemicals to keep food items fresh, reduce costs and maximize profits. In addition, vendors stated that they were not involved with the process of food adulteration. Vendors from the wet market reported that they sell fish immediately after purchase from businessmen rather than holding fish in storage. They collect fish in the morning each day then sell these immediately (Fig. 3). In contrast, staff from the supermarket collect the fish at least a day before displaying it for sale.

A customer from the wet market said,

“When I buy fish, I touch the fish. Touch a fish on its mid-section and if there is resistance, the fish is good. All people know that rotten fish start going soft around the belly. In Dhaka city, vendors simply collect the fish a long time ago and if the fish are not rotting it means they have been drenched in formalin (one kind of chemical)”*.*

Supermarket staff stated that before displaying fish for sale they performed formalin tests according to instructions in the formalin testing kit. In addition, they mentioned that sometimes customers request the sales staff to perform a formalin test in front of them (1–2 customers every day) and it takes 10–20 minutes.

A customer from the supermarket said,

*“There are no foods you can find without adulteration. I know formalin is used in fish, vegetables, and fruits. Day by day there is an increasing rate of food adulteration as we watched it on TV programs and heard from neighbors, relatives. Sometimes newspapers/Facebook published articles regarding food adulteration with toxic artificial* colors. *Moreover, anxiety has increased due to increasing rate of adulterated foods which has negative impacts on health”.*

A customer from the wet market said,

“I am sure that all we eat contains poison, deadly dangerous substances which have negative impacts on human health. We also buy fruits for children: bananas, apples, grapes, oranges, aside from baby foods and milk. Due to the use of particular substances (like-formalin), all foods are fresh and shiny to look at, but you know each fruit is a source of poison.”

Some customers said that shopkeepers try to get the better of them. Half of the wet market customers (*n* = 6) suggested that after picking out the items, they watch closely, to ensure that vendors do not substitute chosen items with something inferior e.g., a sick or smaller chicken, rotten vegetables, or fruits. When customers want to buy vegetables or fruits, they select items that they feel are fresh (*tatka*) and not old or rotten (Fig. 4).

### Customers’ perceptions on health effects, risks of adulterated food, and food safety

Participants were unaware of the magnitude of food adulteration-related deaths in Bangladesh. The majority (*n* = 34) agreed that food adulteration was harmful to one’s health. Half of the customers (*n* = 14) and the majority of participants opined that adulterated foods have long-term adverse effects on health- and might reduce life expectancy. Moreover, participants felt that adulterated foods can have immediate effects e.g., diarrhea, dysentery, and vomiting. Only a few customers from the wet market (n = 3) were aware of the health impacts of adulterated foods.

A customer from the supermarket said,

“In our country, food adulteration is a burning problem which is increasing rapidly day by day. Adulterated foods are dangerous to human health, which leads to increased number of kidney, diabetes, heart attack, and liver failure patients. The respective department of the government should take immediate action against food adulterers to minimize the graveyard situation”

A 30-year old participant from the wet market said;

“In spite of knowing these reasons we do not have any alternative without having these foods. This should be taken as public health urgency, not only by Government but also take the initiatives by NGOs”

Most participants (*n* = 34) stated that no food can be regarded as safe. A few participants reported that safe food denotes food that is free from any kind of adulteration or free of formalin. In addition, more than half of the customers perceived formalin residue to be a serious food safety threat that could impact health; this issue has been repeatedly raised by the local press.

### Factors influencing food purchase

**Fig. 2:**
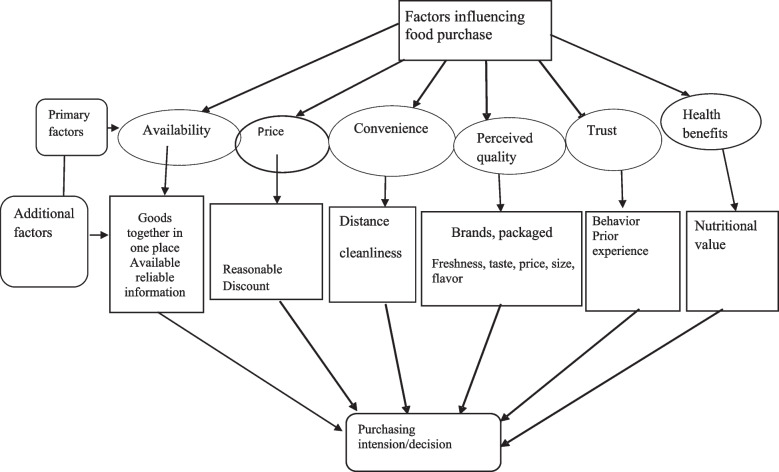
Factors influencing food purchase

Various factors that influence customers’ food purchasing behavior were identified during interviews and discussions and synthesized into a conceptual model (Fig. 2), used to summarize data in this section.

While participants preferred unadulterated foods for a healthy life, with little information on safety and safe alternatives, they purchase based on their usual pattern of consumption, which they said was difficult to change, highly cultural, having formed during one’s early years. Consequently, choices are made primarily on price, convenience, perceived quality, and trust.

A customer from the wet market said;

“We are bound to eat to survive. No one can take care of this issue [food adulteration]; we are helpless as we do not have any alternative without buying those.”

#### Price

Participants from the lower-income group, who struggle for their subsistence, try to purchase food at a low price and are less concerned with food safety or quality. In contrast, participants from the higher socio-economic group pay more at supermarkets compared to the wet market. Customers from group discussion (*n* = 6) argued that vendors are more concerned about the price of the goods than quality or adhering to the recommended price. Price was the most frequently mentioned priority for participants from wet markets. Customers from both locations mentioned that prices are generally higher at the supermarket. Supermarket customers were motivated by the price discount system and purchasing at a fixed price, with no bargaining necessary. In addition, in some cases, customers thought that they pay an additional cost, estimated at USD 0.10 per kilogram, in the supermarket compared to the wet market.

#### Convenience

The wet market customers mentioned that the key priority was that the market was close to their house (5–10 minutes walking distance). Conversely, supermarket customers said that though the location was distant from their homes and involved additional transport costs, they liked the supermarket’s cleanliness and the convenience of obtaining all goods at one location and transaction.

A customer from the wet market said;

“My father purchased foods from the wet market since 1983 when I was 8 years old. My grandfather also purchased foods from the same bazaar and same vendor. So, to me, the relationship with the vendor is the key point which could motivate me to purchase foods, along with the location of market as I need only five minutes to go there.”

#### Food quality; dry goods

Most participants (*n* = 8) from the supermarket mentioned their prime concern was food quality, and they determined the quality of foods in terms of packaged item brands, size, perceived freshness, taste, and flavor. Also, they expressed that they trusted quality by examining food labels on packaged goods. Food labeling frequently includes the brand name, total weight, ingredients, and amount (s) including nutrient values, date of manufacture, and expiry, which had an important role in determining perceived food quality, stated by one-third of the supermarket customers. In addition, in supermarkets, they checked product quality on arrival and disposed of unacceptable/decomposed vegetables, fish, and or meat. More than half of the customers from the supermarket said that it stocked good quality foods compared to wet markets.

The wholesalers from the wet market reported that they sell rice from jute sacks and *polyethylene* bags where rice is labeled as ‘export quality,’ ‘fortified with nutrients,’ ‘stone free’, as written on the outside of the bag or sacks by producers. They said that rice producers consider these as quality indicators whereas customers do not make these distinctions. Moreover, vendors from the wet market described that attractive packaging and labeling impacts customers’ perception of food quality, especially freshness and taste. Only a few customers (7/38; *n* = 4 from supermarket and *n* = 3 from wet market), mentioned nutritional value. While purchasing rice, two customers and one vendor mentioned the brand or company name. Customers from the supermarket also considered expiry date, quality, and freshness as important criteria while buying packaged food items; only a few (n = 3) considered the packaged instructions-brand name, ingredients (*n* = 2), percentage of nutritional values (*n* = 1). Some from the wet market said that they bargain to get a good item. None mentioned that they looked for the approval of the regulatory authority- *Bangladesh Standards and Testing Institution* (BSTI) when buying packaged and regular food items. A few mentioned that when they wanted to purchase the items quickly in case of urgent need they purchased from the supermarket.

Among customers from both locations, there were frequent comments on food quality.

A vendor from the wet market said;

“I try to make sure that no medicine or powder was used to store rice. Although I do not know the name of those medicines or powders, I tried to ensure the quality of the food. I have heard from my acquaintances that those are harmful to health.”

A customer from the supermarket said,

“To me, supermarket and the wet market are the same things considering the quality of foods. The supermarket has no arrangement for test of foods, supply fresher foods. So how can I realize if the food in supermarket is good, and the food in the wet market is free of adulteration?”

#### Food quality; fresh foods/perishables

**Fig. 3:**
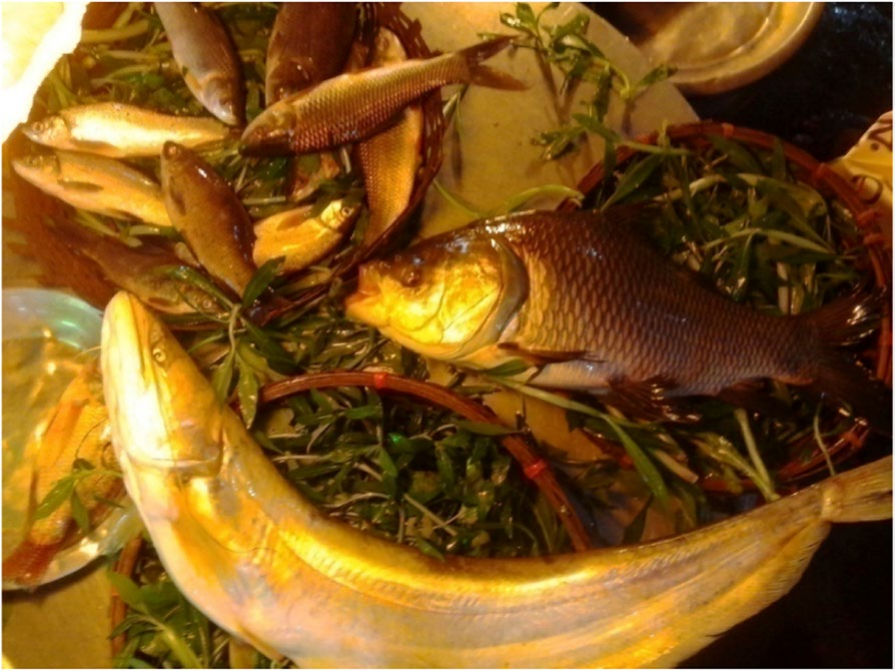
Fish displayed at the wet market with greenery to look more ‘natural’ and ‘fresh’

**Fig. 4:**
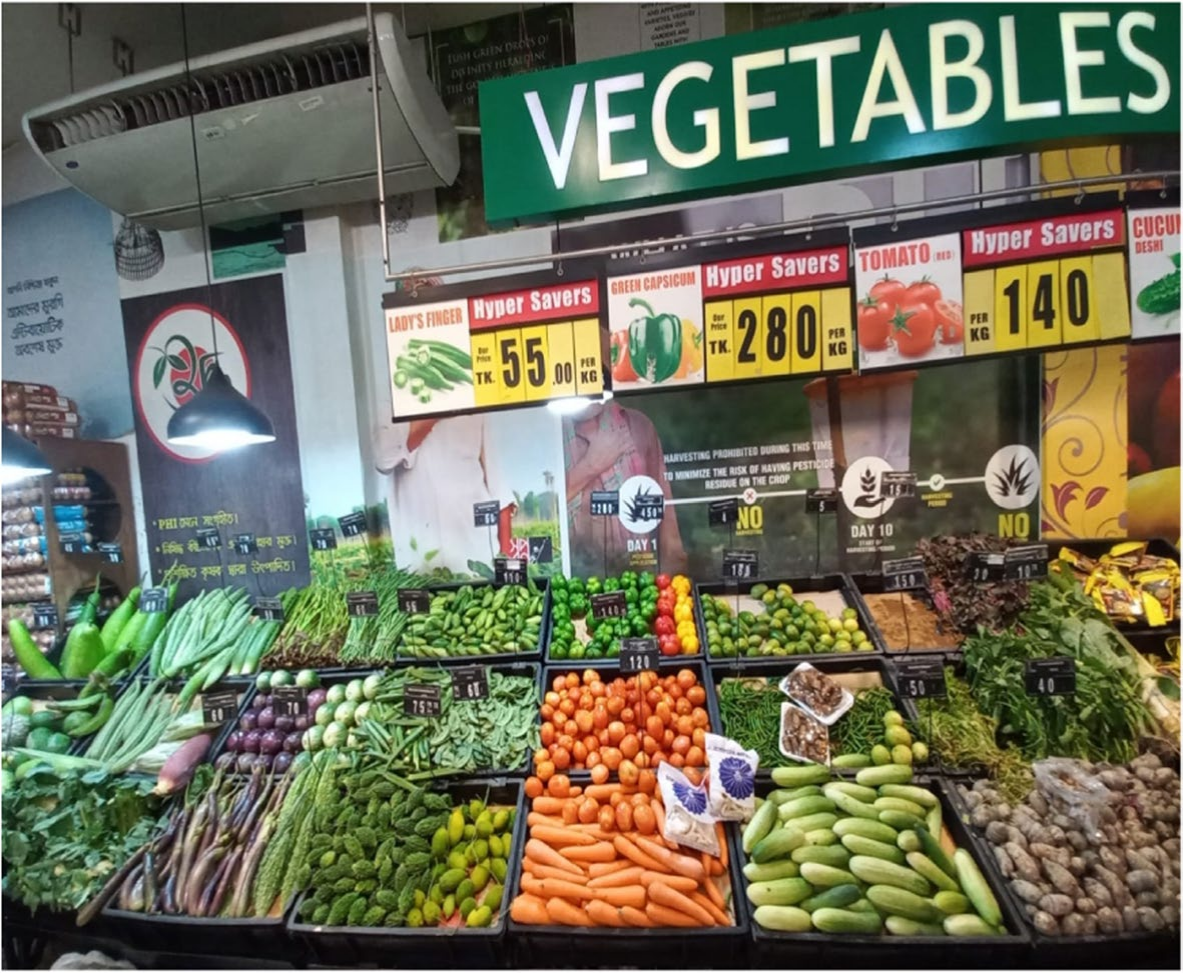
Vegetables displayed at supermarkets

Fish quality was judged by customers from both markets and by vendors from the source (geographical region and whether it was from the river or sea). In contrast, vendors from the wet market stated that they do not need to check the quality of foods. Usually, they collect fresh foods-vegetables, fish, meat each day and sell these immediately. Only in some exceptions, when they have residual foods such as fish or meat in their shops do they store and sell the next day. Nearly all (*n* = 10) customers from the wet market and two vendors reported that food quality was ascertained by touching and observing goods. Customers also observed fish at the wet market and found it displayed with greenery that made it look more ‘natural’ and ‘fresh’ (Fig. 3). In addition, one-third of the supermarket customers and three customers from the wet market reported that some foods need to be cooked first to understand the quality e.g., rice, fish, and meat (Fig. 4). Therefore, customers perceived that food quality was a mediating factor for purchasing behavior.

A customer from the supermarket said,

“*Quality does not differ a lot. I purchase these local products like vegetables from other markets or farms as I have no own products. The quality of green vegetables is looking similar everywhere. A wet market is enriched with different green vegetables, and they collect these regularly which is fresher compared to the supermarket. A supermarket is superior due to its cleanliness in some sense. It’s a matter of fact and one of the reasons which could make difference between supermarket and wet market.”*

A vendor from supermarket said,

*“If anyone thinks fish is a healthy option, they should think about it again. Many fish vendors spray formalin on fish (a chemical usually used for the preservation of fish for a longer period). This kind of chemical is usually used with fish including imported fish. It makes the fish stiff and keeps them looking fresh for a longer duration”.* (

**Fig. 5:**
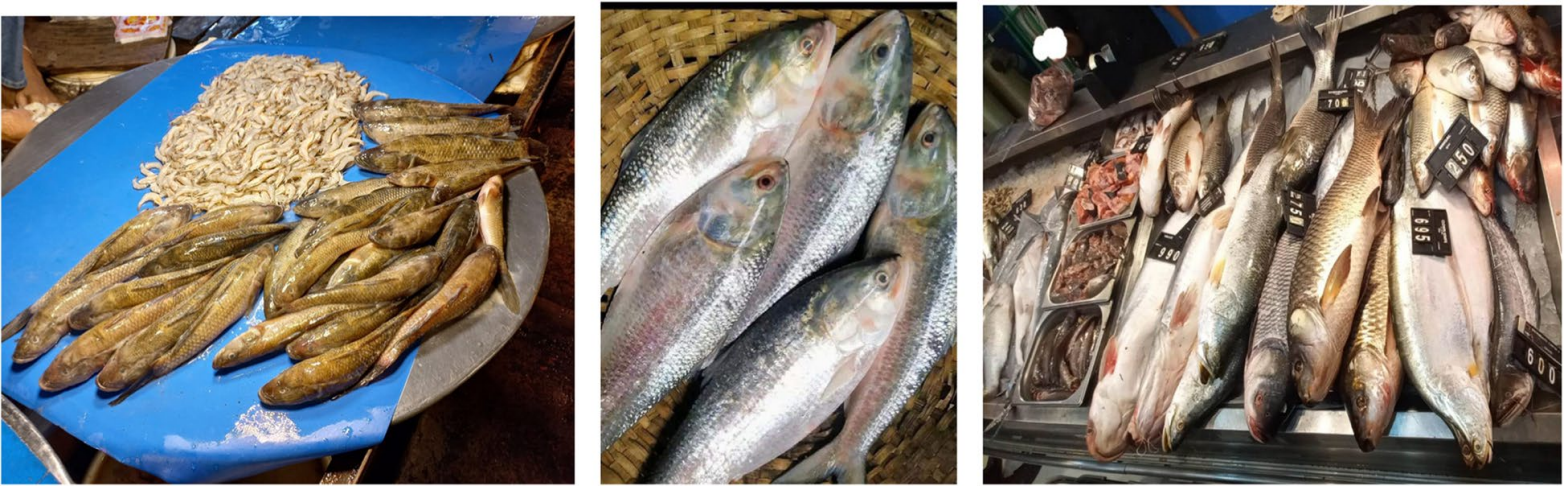
Fish perceived as formalin tainted (left to right: wet market-photo-1, 2 & supermarket-photo-3)

Fig. 5)

In contrast, another participant from the wet market said,

“Whenever I purchase some vegetables, like tomato, green chili, pepper, from the wet market they remain fresh day after day without getting rotten but in the supermarket, it’s found rotten after one day. I perceived getting rotten vegetables fast as a good sign which is free of formalin. It’s a matter; I think that the sellers, who sell at the wet market, use chemicals and items that stay dry and don’t rot. But in the case of the supermarket vegetables become rotten.”

#### Trust in vendors

Study participants reported that a customer’s intention and decision to purchase foods can be driven by vendor trust and in most cases, particularly in the wet market, vendors get return customers when they feel trusted. Personal acquaintance with the vendor leads to trust as customers can be motivated by vendors’ behavior. Supermarket customers said that the supermarket staff is educated and interactions with customers are good. In addition, they have a minimum level of understanding related to dealing with a customer. Staff behavior is an important consideration for supermarket customers.

A customer from the wet market said,

“I purchased rice from a vendor in Mirpur-2 market. I trust him as I am purchasing rice from his shop since 2010. However, when I purchased rice first time for my family, I relied on his opinion. I have no idea about good quality of rice. When rice was been cooked, my family members said that the quality of rice was good. There are some items which are not possible to ensure quality without cooking or eating.”

“Vendors of the wet market do not store the food items like vegetables, fish, meat for selling on next days, but in the supermarket it happens, and they usually do it.”

A customer from the wet market said,

“The vendors are familiar to me as I have been shopping from this market for a long time around 14-15 years; to me, trust of the vendor is important for getting quality foods

A customer from the supermarket said,

“I have purchased consumable foods from this supermarket since 2014. Almost all staffs are familiar with me. I do not know what are quality food or fresh fruits or vegetables, but they provided me the accurate items and fresh ones. So, I relied on their trust. Also, their behavior motivated me to purchase foods from this shop.”

A customer from the wet market said,

*“All times I am purchasing Rashid (brand)* minicat *(variety) rice as I know it is the renowned company and the rice of this brand is good-clean compared to others. Since 2009, I and my family members prefer* minicat *(variety) rice and I purchase rice only from the vendor of the open-air market of Mirpur-6. Even there I did not find any extra substances in the rice such as stones, black rice grains. So, I believe the vendor keeps the best quality of rice in his shop.”*

### Customer awareness and availability of reliable food information

We found that most (*n* = 32) participants were aware of food adulteration; among them, most were male customers (*n* = 25) and a few female customers (*n* = 7) were aware. Most participants (*n* = 31) opined that food adulteration had become more common and felt that this problem persists at all levels of the food chain from preparation to consumption.

A market committee member from the wet market said*,*

“An overall customer perception of food adulteration is high. I think if anyone talks about adulterated foods whereas about eight in ten people in each market describe the impacts of adulterated foods in people’s daily life. Food adulteration in Bangladesh is highly prominent without any concern of health aspects of foods.”

Half of the participants (customers and vendors, *n* = 19) stated that educated people were more concerned about food adulteration. About half of the customers and vendors (*n* = 17) reported that there is a law in the country that deals with food adulteration; among them, nine participants thought that the existing law and enforcement was insufficient. In addition, some mentioned that monitoring adulterated foods is not strong.

Despite purposively enrolling participants from the two shopping venues to maximize variability in responses, we found that those from the wet market and the supermarket had similar and limited access to reliable customer information about food adulterants. One-third of the participants (*n* = 12) from interviews and half of the participants from group discussion mentioned that though they do not have access to reliable information, they are concerned about food adulteration. Television, family members, newspapers, other media, Facebook, friends, neighbors, colleagues, fellow customers were the reported sources of information. Most of the participants (*n* = 24) reported that they shared information with others if they found any adulterated food item while the remainder (n = 12) did not share information.

A participant from the wet market said;

“My acquaintances have only told about the adulterated food; they do not know anything else in detail. Could you guide us in which place and from whom mass people can learn the information related to adulterated food?”

### Consumers’ perceptions and attitudes about testing adulterated food

Study participants from both markets had similar opinions about the testing of adulterated food. They perceived severity of adulterants in food and acceptance of scientific uncertainty about the risk. Additionally, the perception of adulterated food varied between participants in terms of the degree of chemicals being harmful and correlation with adulterated food and perceived health risk.

More than half of the participants in interviews (*n* = 17) and one-third of the participants from the group discussion (*n* = 3) distinguished between fake food and adulterated foods.

A customer from the supermarket said,

“I heard from my colleague that plastic rice is available in the market came from China. So, what can we do? Who can take care of this? We are helpless because of the fake food items. We are facing many health-related problems?”

A customer from the wet market said,

“I saw news on Facebook that artificial egg is available in the market. It was made by using multiple chemical substances which are dangerous for human health. If anyone could identify the artificial egg (s)/he should not buy it surely.”

## Discussion

Almost all study participants contended that most foods are adulterated and that there is a public health threat from exposure to dangerous toxins and artificial colors, similar to studies from India [14, 22]. In this qualitative study, we explored consumers’ assessment of uncertain risks of chemical substances in food. None of the participants in this study volunteered a source of information on chemical adulteration to inform risk perception. Perceptions about risk were therefore formed on the spot, based on prior knowledge and beliefs about chemical risks in general and food safety [38].

This is the first qualitative study from Bangladesh to include supermarket customers that investigate participants’ understanding/perception of adulterated foods, and appraisal of the risk of contaminating substances for different foods. Participants perceived that adulterant levels in foods were excessive. Although there are laws on food adulteration (Food Safety Act, 2013) and a responsible authority (BSTI), enforcement was considered insufficient. Participants from both markets estimated that around 70% of food items were commonly adulterated with harmful chemicals or artificial colors, similarly found in a cross-sectional survey among residents of Dhaka city, Bangladesh that reported more than 35 food items were commonly adulterated [23]. In line with existing literature [39] participants acknowledged the harmful health effects of chemical substances in food. Together, these findings support the view that the public evaluates hazards and risks differently when the risks are undetermined [38], and that a trade-off between appraisal of the hazard and appraisal of the risk may guide consumer responses.

Moreover, we found that supermarket customers seemed more concerned about adulterated foods than wet market customers. Customer adulteration concerns were described by supermarket staff as requests for on-the-spot formalin testing, a facility that is not available at wet markets. The study participants from both markets perceived that the overall proportion of food adulteration was increasing, in contrast to a previous study from 2001 to 2005 which identified a perceived decrease [23].

Customers saw no alternative to purchasing adulterated foods. They stated that vendors were more concerned about the price of goods than quality. Price was the most frequently mentioned consideration among customers affecting buying intention. Participants from the supermarket mentioned their prime concern was food quality as a mediating factor for purchasing behavior. Among the factors affecting purchase decisions, convenience was important for participants from both markets; wet markets attracted customers closely located and the compact location with prepackaged foods provided convenience for supermarket customers. The role of convenience in customers’ food preferences was explored in a recent study [40]. Other factors affecting purchase included freshness, brand, availability of packaged items, taste, size, flavor, cleanliness, and market location.

Most customers were aware of the risk of consuming adulterated foods and related subconscious values and beliefs to their preferences and food choices, similar to other reports [41, 42]. In this study, customers decided to purchase specific foods based on perceived quality taste, texture, religion, and nutritional values, as found previously [40, 42, 43]. We found that food quality and food safety is driven by several factors similarly reported in other studies. Freshness was the most frequently cited factor following price and cleanliness when participants thought about the quality of food [11, 42, 44]. In addition, our study found that participants determined the quality of foods based on prior experience; customers assessed quality after cooking and consumption. At the point of purchase, in some instances, customers touched food to determine freshness, while others inspected packaging for expiry dates and nutritional value.

Regardless of gender, household income, and educational status, participants reported that they had limited access to reliable, trustworthy information on adulterated foods, needed to support food purchase choices based on optimal food safety. They expressed the need for access to credible information about food adulteration. In recent years, similar to other studies in Bangladesh, we found that substantial resources have been devoted to increasing the level of food safety perception and knowledge among customers [22, 45]. The study indicates that the media, mostly newspapers, television, and Facebook are the main sources of food adulteration information for customers.

Although laws and government initiatives against those who are responsible for food adulteration exist, there is limited capacity for identifying adulteration and taking action. In addition, the food safety authority likely lacks the technical expertise and the considerable financial and institutional investment to systematically test and report food adulteration. Despite this, the government was reported by participants as a source of trustworthy information. To develop a preventative strategy, an effective food safety regulatory framework, financial and institutional investment is needed to reduce food adulteration. This would be a significant undertaking for a LMIC government, and it would be costly.

This study focused on participants from two urban markets that were geographically concentrated in Mirpur, Dhaka, Bangladesh. People from other parts of the country, especially from rural areas, may have different perceptions. Therefore, findings may be limited in generalizability or may not represent all urban dwellers, or country-wide perceptions of food adulteration. Nevertheless, participants’ perceptions identified important gaps in the food safety system.

## Conclusion

Participants perceived food adulteration as a health concern and that unadulterated foods were rare and purchased based on price, quality, taste, flavor, market location, cultural preferences and convenience. They would like results from a government testing program to act as a source of trustworthy food adulteration information. Baseline data on the content and extent of adulterated foods could inform subsequent strategies to reduce adulteration and consequently health threats. To advocate government leadership to reduce food adulteration, credible population-based data on disease burden, a model food sampling and testing protocol, a model for inspections, example social and behavioral change communication with associated costs that include organizational strengthening and training are needed.

## Data Availability

The datasets used and/or analyzed during the current study are available from the corresponding author on reasonable request.
